# Redrawing the US Obesity Landscape: Bias-Corrected Estimates of State-Specific Adult Obesity Prevalence

**DOI:** 10.1371/journal.pone.0150735

**Published:** 2016-03-08

**Authors:** Zachary J. Ward, Michael W. Long, Stephen C. Resch, Steven L. Gortmaker, Angie L. Cradock, Catherine Giles, Amber Hsiao, Y. Claire Wang

**Affiliations:** 1 Center for Health Decision Science, Harvard T.H. Chan School of Public Health, Boston, Massachusetts, United States of America; 2 Department of Prevention and Community Health, Milken Institute School of Public Health, the George Washington University, Washington, District of Columbia, United States of America; 3 Department of Social and Behavioral Sciences, Harvard T.H. Chan School of Public Health, Boston, Massachusetts, United States of America; 4 Department of Health Policy and Management, Mailman School of Public Health, Columbia University, New York, New York, United States of America; Hospital Universitario LA FE, SPAIN

## Abstract

**Background:**

State-level estimates from the Centers for Disease Control and Prevention (CDC) underestimate the obesity epidemic because they use self-reported height and weight. We describe a novel bias-correction method and produce corrected state-level estimates of obesity and severe obesity.

**Methods:**

Using non-parametric statistical matching, we adjusted self-reported data from the Behavioral Risk Factor Surveillance System (BRFSS) 2013 (n = 386,795) using measured data from the National Health and Nutrition Examination Survey (NHANES) (n = 16,924). We validated our national estimates against NHANES and estimated bias-corrected state-specific prevalence of obesity (BMI≥30) and severe obesity (BMI≥35). We compared these results with previous adjustment methods.

**Results:**

Compared to NHANES, self-reported BRFSS data underestimated national prevalence of obesity by 16% (28.67% vs 34.01%), and severe obesity by 23% (11.03% vs 14.26%). Our method was not significantly different from NHANES for obesity or severe obesity, while previous methods underestimated both. Only four states had a corrected obesity prevalence below 30%, with four exceeding 40%–in contrast, most states were below 30% in CDC maps.

**Conclusions:**

Twelve million adults with obesity (including 6.7 million with severe obesity) were misclassified by CDC state-level estimates. Previous bias-correction methods also resulted in underestimates. Accurate state-level estimates are necessary to plan for resources to address the obesity epidemic.

## Introduction

Overweight and obesity are among the leading causes of morbidity and mortality in the United States [1,2]. The adult state-specific obesity maps developed by the Centers for Disease Control and Prevention (CDC) highlight the magnitude of this problem, as well as the large disparities that exist by state [3]. These maps and related local prevalence data have galvanized state leaders to take action, and have been used to prioritize federal obesity prevention resources [4].

However, despite the alarmingly high obesity rates depicted in recent CDC maps, these figures may substantially underestimate the true state-level burden, as they rely on self-reported height and weight data from the telephone-administered Behavioral Risk Factor Surveillance System (BRFSS) [5]. Bias in self-reported body measures is well-documented [6], and results in underestimates of body mass index (BMI, kg/m^2^). Data from in-person interviews reveal that on average, women underestimate their weight by about 1 kg, and adults in general overestimate their height by about 1 cm (see Table A.1 in S1 File); similar biases exist for telephone respondents (see Table A.2 in S1 File). These relatively small individual-level biases can result in large differences for population estimates—especially since height is squared to calculate BMI.

Nationally, obesity prevalence based on self-reported data from BRFSS 2013 was 29%, in contrast to 34% using objectively-measured height and weight data from the National Health and Nutrition Examination Survey (NHANES). While NHANES is useful for monitoring national trends in obesity, its relatively small sample size (and lack of data collection in every state during each survey) is insufficient to produce yearly state-specific estimates of obesity prevalence [7]. As a result, no nationally-representative, objectively-measured BMI surveillance system exists that can provide unbiased estimates of state-specific obesity prevalence. This lack of accurate data limits states’ ability to evaluate the health and economic effects of the obesity epidemic and to plan preventive policies and programs.

Previous efforts to address self-report bias have used regression models to analyze the relationship between self-reported and measured height and weight data from NHANES [8–11]. However, we show that these approaches underestimate obesity prevalence compared to objectively measured estimates. We describe a novel method of bias correction using non-parametric statistical matching to combine all available data to generate more accurate estimates of the entire BMI distribution. We compare the obesity prevalence results from our method to uncorrected estimates, and to regression-based approaches to bias correction [9,11–13].

## Methods

### Statistical Matching

We developed a non-parametric statistical matching algorithm [14–16] to adjust state-specific, self-reported height and weight from BRFSS 2013 (n = 386 795) using the relationship between self-reported and measured data from individuals in NHANES 2007–2012 (n = 16 924). Statistical matching combines data from separate datasets (i.e. BRFSS and NHANES) that are based on the same underlying population (i.e. non-institutionalized civilian adults in the US aged 18 and older), but that do not have an individual identifier in common [15]. It has been used in fields such as economics, ecology, health, and social policy to synthesize comprehensive datasets from a range of sources [17–21]. One advantage of this approach is the preservation of the marginal distributions of imputed variables from the underlying datasets. This allowed us to maintain the measured national distribution of BMI from NHANES while incorporating the self-reported state-level variation from BRFSS.

The statistical matching algorithm was developed as part of the CHOICES (Childhood Obesity Intervention Cost-Effectiveness Study) project, a larger model-based initiative in which the US population is simulated to evaluate a range of obesity prevention policies and programs. We developed the model in Java, an object-oriented programming language.

### Datasets

The Behavioral Risk Factor Surveillance System (BRFSS) is a nationally-representative telephone survey of adults which completes more than 400 000 interviews each year and is the foundation of the CDC obesity prevalence maps [22]. BRFSS collects data from US residents regarding their health-related risk behaviors and self-reported height and weight. We used survey data from 2013 which had 491 773 responses. After ensuring that no data were missing for demographic variables of interest and self-reported height and weight (n = 102 339), and after excluding pregnant women because of possible effects on weight (n = 2639), data for 386 795 individuals remained.

The National Health and Nutrition Examination Survey (NHANES) assesses the health and nutritional status of adults and children, and is unique in that it is the only ongoing national survey of adults that has both self-reported and measured height and weight [7]. In 1999 the survey became a continuous program and examines a nationally representative sample of about 5000 people each year. We pooled NHANES data from 2007–2012, which included observations from 18 619 adults. After excluding pregnant women (n = 182) and respondents missing data for the variables of interest (n = 1513), the final sample included 16 924 respondents aged 18 and older. Pooled sample weights were calculated following the NHANES analytic guidelines [23]. The complex survey designs were taken into account for both BRFSS and NHANES.

We re-categorized race/ethnicity and household income to ensure that these variables had common definitions across the BRFSS and NHANES datasets (see Table B in S1 File). Due to its smaller sample, NHANES has more limited detail on race/ethnicity compared to BRFSS. In order to make the datasets comparable, we included individuals in the BRFSS dataset who reported their race/ethnicity as “American Indian or Alaska Native”, “Asian”, “Native Hawaiian or Other Pacific Islander”, “Other”, and “Two or More Races” in the “Other” category in the matched dataset. While the latest round of NHANES (2011–2012) does include a race/ethnicity code for “Asian,” we coded this category as “Other” so that these data could be combined with previous waves of NHANES. For estimates in Hawaii, matching was performed across all races for non-Black minorities to avoid biasing the BMI distribution by failing to distinguish between Native Hawaiian and Asian individuals.

### Matching Algorithm

Individuals in NHANES and BRFSS were matched by national-level percentiles of self-reported height and weight within demographic subgroups (defined using age, sex, race/ethnicity, and income) with probability proportional to their sample weight [24]. Measured values of height and weight were obtained for each sampled NHANES individual, and up to 1% of random variation was added in order to smooth the distributions [25]. Because the same subgroups were used across datasets, we controlled for differences in demographic composition, thus estimating the state-level geographic effect on obesity within subgroups. This approach also controlled for any differential self-report bias of height or weight by age, sex, race/ethnicity, and household income.

Although matching can be done with greater precision within tightly-defined subgroups, a balance must be sought—over-stratifying the matching may fail to preserve heterogeneity in the synthesized joint distribution, and may lead to no possible matches. On the other hand, defining the subgroups too loosely may lead to inappropriate matches. To address this issue, we used dynamic subgroup definitions contingent on a minimum sample size, which we varied empirically to yield the desired balance between sample heterogeneity and matching precision. Specifically, we used age- and sex-specific thresholds that yielded BMI distributions statistically similar to NHANES. These thresholds were selected using a grid search that minimized the maximum distance between the cumulative distributions. If the subgroup sample was below the specified size, the matching restrictions were gradually loosened until the threshold was met (see Table C in S1 File). Within subgroup samples, percentile-matching bandwidths were initialized to zero and expanded in a similarly iterative way until a match was found.

Since matching is a stochastic process [14,15], in order to explore uncertainty and arrive at stable estimates, individual-level BMI in the final dataset was calculated using the mean adjusted values over 100 iterations of the matching process. Sample-weighted state-level estimates of the BMI distribution and the prevalence of obesity (defined as BMI≥30 kg/m^2^) and severe obesity (defined as BMI≥35 kg/m^2^) were then calculated, accounting for the survey design in the original BRFSS dataset.

### Model Comparison

We compared the statistical matching method to previously published approaches to bias correction. A method described by Cawley [9] uses individual-level regression models comparing self-reported to measured heights and weights within NHANES. An alternative approach described by Dwyer-Lindgren [11] regresses aggregate-level estimates to align self-reported mean BMI with the measured mean from NHANES. This approach forms the basis of obesity maps hosted by the Institute for Health Metrics and Evaluation [26]. For direct comparability, we re-estimated these models with our datasets (see Tables D and E in S1 File). We evaluated the bias-corrected BRFSS datasets from all methods against the measured BMI distribution and prevalence of obesity and severe obesity from NHANES. The adjusted prevalence estimates were compared to NHANES using χ^2^ tests, and the adjusted age/sex-specific BMI distributions were compared to the distributions from NHANES using two-sample Kolmogorov-Smirnov tests—a non-parametric, distribution-free test sensitive to differences in the location and shape of cumulative distributions [27].

## Results

Estimates of mean BMI for US adults were similar for both the statistical matching and regression-based methods. Whereas uncorrected BRFSS data yielded a mean BMI of 27.77 (95% CI, 27.73–27.81), the individual-level regression model estimated 28.40 (95% CI, 28.36–28.45), the aggregate regression model estimated 28.53 (95% CI, 28.49–28.57), and statistical matching yielded 28.49 (95% CI, 28.45–28.54). In comparison, the mean BMI in NHANES was 28.50 (95% CI, 28.30–28.69).

Although mean BMI was similar for the adjustment methods, obesity prevalence was not. Based on χ^2^ tests, only the statistical matching method yielded estimates of obesity and severe obesity that were not statistically different from NHANES (see Tables 1 and 2). Using recent census population counts [28], the corrected obesity prevalence corresponded to approximately 81 million adults with obesity in the US, of whom 33 million had severe obesity (BMI ≥35). In contrast, the 2013 CDC map underestimated the size of the population with obesity by 12 million (including 6.7 million cases of severe obesity). Regression-adjusted estimates also underestimated adult obesity prevalence by 1.8–3.2 million people, and severe obesity prevalence by 1.3–1.4 million.

**Table 1:** Comparison of obesity prevalence (BMI≥30) by method to measured data from NHANES.

Sex	Values	NHANES	BRFSS (Uncorrected)	Individual-level Regression^a^	Aggregate-level Regression^b^	CHOICES Model (Statistical Matching)
**Overall**	**Prevalence % (95% CI)**	34.01 (32.71–35.31)	28.67*** (28.38–28.96)	32.47*** (32.16–32.77)	33.02** (32.72–33.33)	33.79 (33.48–34.10)
	**χ**^**2**^ **(*P* Value)**	---	225.0 (*P* < .001)	17.5 (*P* < .001)	7.2 (.007)	0.4 (.55)
**Males**	**Prevalence % (95% CI)**	33.03 (31.33–34.73)	28.43*** (28.00–28.85)	31.58** (31.14–32.02)	32.02 (31.57–32.46)	32.43 (31.99–32.87)
	**χ**^**2**^ **(*P* Value)**	---	83.5 (*P* < .001)	7.8 (.005)	3.8 (.052)	1.3 (.25)
**Females**	**Prevalence % (95% CI)**	34.96 (33.48–36.45)	28.92*** (28.52–29.32)	33.38** (32.96–33.80)	34.06 (33.64–34.48)	35.20 (34.77–35.62)
	**χ**^**2**^ **(*P* Value)**	---	143.8 (*P* < .001)	9.1 (.003)	2.9 (.087)	0.2 (.66)

^a^ Individual-level regression method is based on models by Cawley et al. [9]

^b^ Aggregate-level regression method is based on models by Dwyer-Lindgren et al. [11]

Asterisks indicate level of statistical signifance:

*** *P* < .001,

** *P*< .01,

* *P* < .05.

**Table 2:** Comparison of severe obesity prevalence (BMI≥35) by method to measured data from NHANES.

Sex	Values	NHANES	BRFSS (Uncorrected)	Individual-level Regression^a^	Aggregate-level Regression^b^	CHOICES Model (Statistical Matching)
Overall	Prevalence % (95% CI)	14.26 (13.45–15.07)	11.03*** (10.83–11.23)	13.29*** (13.07–13.50)	13.27*** (13.05–13.49)	13.84 (13.62–14.06)
	χ^2^ (*P* Value)	---	170.6 (*P* < .001)	13.2 (*P* < .001)	13.8 (*P* < .001)	2.4 (.12)
Males	Prevalence % (95% CI)	11.48 (10.48–12.49)	9.49*** (9.21–9.77)	11.23 (10.94–11.52)	10.98 (10.68–11.27)	10.79* (10.50–11.08)
	χ^2^ (*P* Value)	---	36.9 (*P* < .001)	0.5 (0.48)	2.1 (.15)	4.0 (.046)
Females	Prevalence % (95% CI)	16.95 (15.91–17.99)	12.62*** (12.33–12.92)	15.41*** (15.09–15.73)	15.64** (15.31–15.96)	16.99 (16.66–17.32)
	χ^2^ (*P* Value)	---	136.9 (*P* < .001)	14.8 (*P* < .001)	10.6 (.001)	0.009 (.92)

^a^ Individual-level regression method is based on models by Cawley et al. [9]

^b^ Aggregate-level regression method is based on models by Dwyer-Lindgren et al. [11]

Asterisks indicate level of statistical signifance:

*** *P* < .001,

** *P*< .01,

* *P* < .05.

Statistical matching performed significantly better than regression methods for age and sex subgroups as well. According to the two-sample Kolmogorov-Smirnov tests, no age/sex-specific BMI distribution produced by our method was statistically different from the corresponding subgroup in NHANES (see Table F in S1 File).

**Fig 1:**
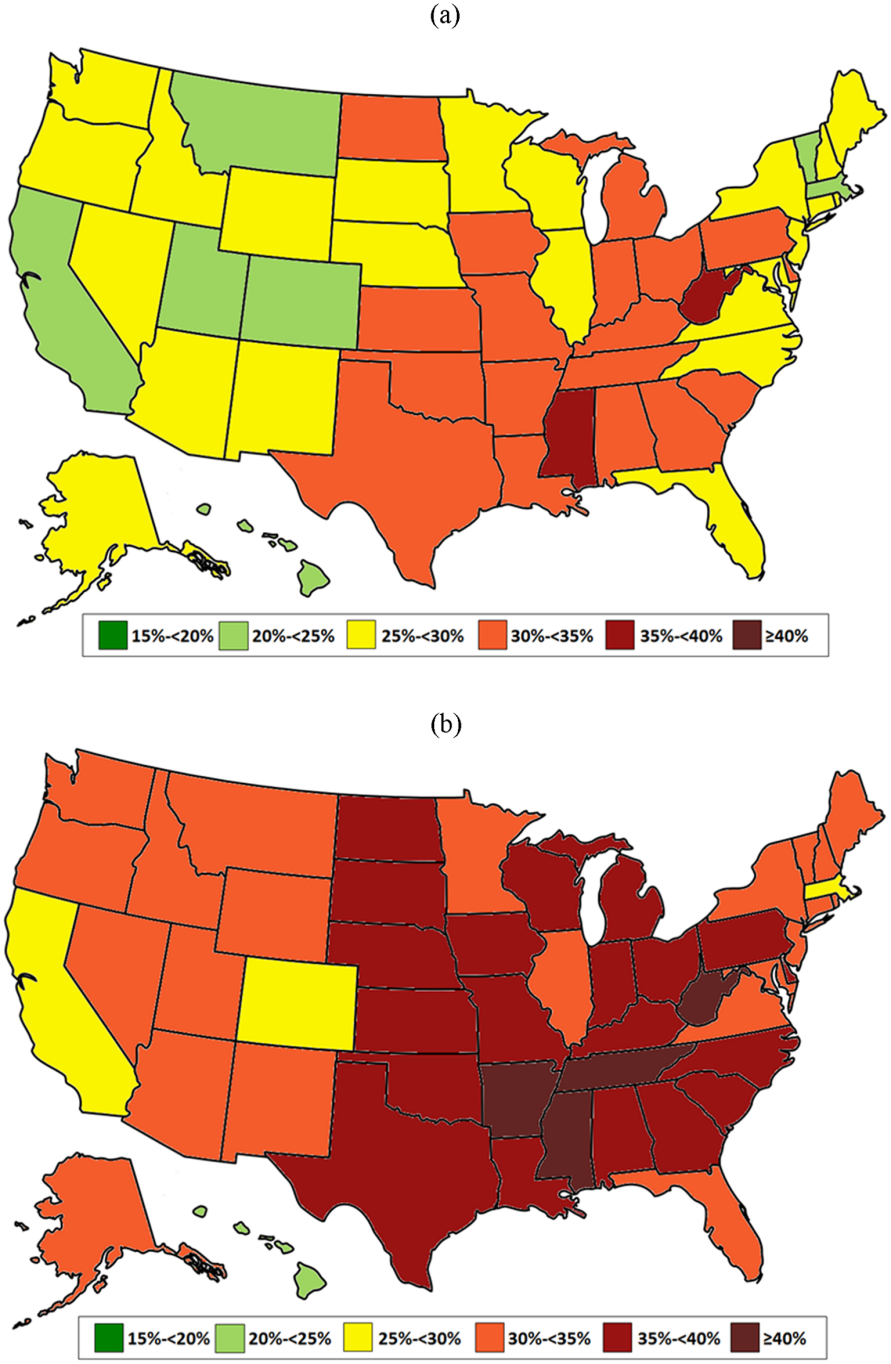
Prevalence of adult obesity (BMI≥30) by state in 2013 –(a) Uncorrected vs. (b) Corrected.

**Fig 2:**
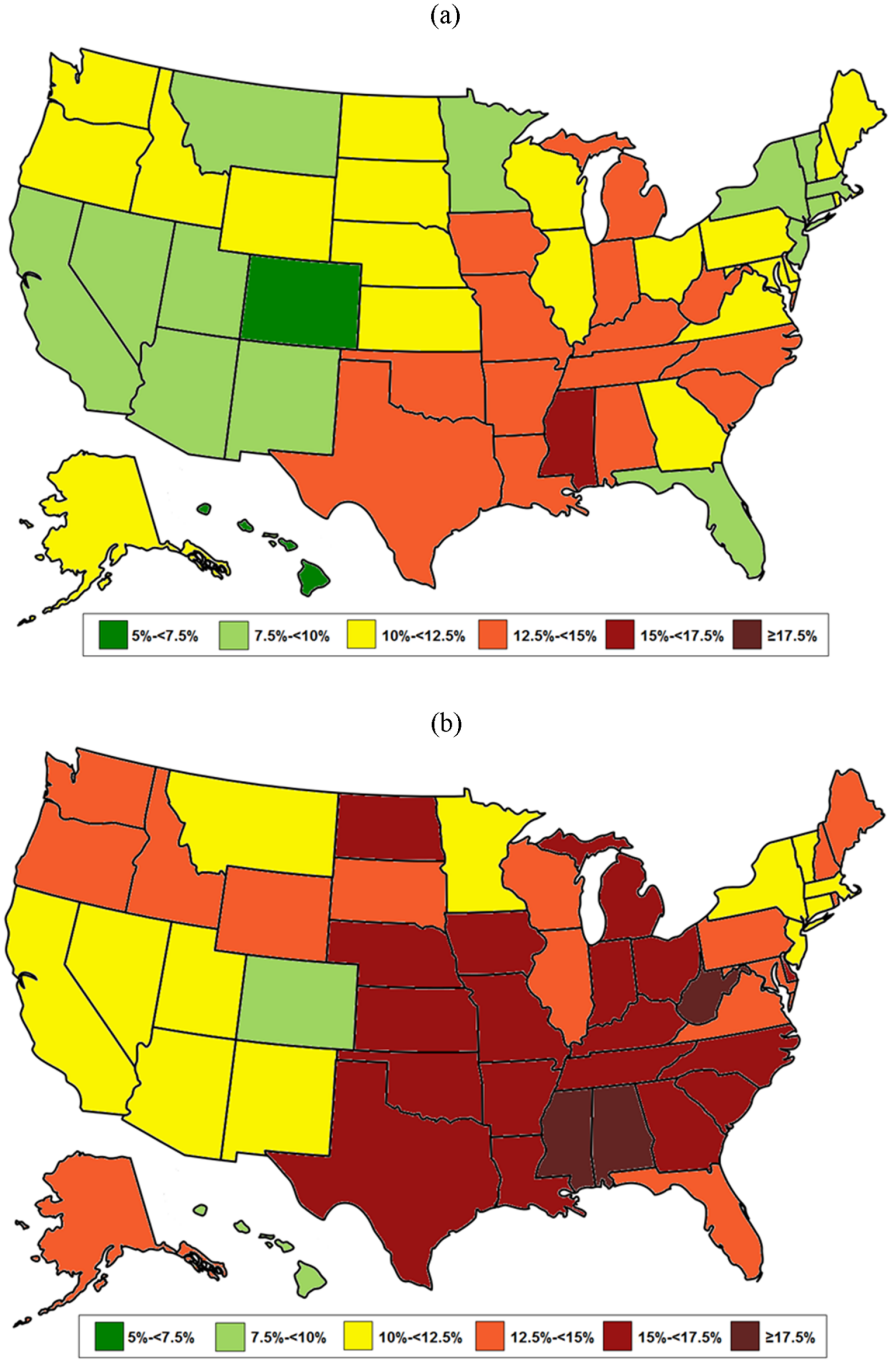
Prevalence of adult severe obesity (BMI≥35) by state in 2013 –(a) Uncorrected vs. (b) Corrected.

Lastly, state-specific obesity estimates for 2013 were compared with the CDC figures [29,30] (Figs 1 and 2 and Table 3). Based on the bias-corrected estimates from statistical matching, only four states (California, Colorado, Hawaii, and Massachusetts) had an adult obesity prevalence below 30%. This contrasts with the CDC map in which a majority of states were below this level. In four states (Arkansas, Mississippi, Tennessee, and West Virginia), the estimated obesity prevalence was over 40%, a category not included in any previous CDC map. A similar picture exists for severe obesity—corrected estimates reveal that 3 states (Alabama, Mississippi, and West Virginia) have a prevalence of severe obesity greater than 17.5%, a level not seen in the uncorrected data.

**Table 3:** Adult obesity prevalence (%) by state in 2013 –CDC versus corrected CHOICES model estimates.

State	Obesity (95% CI) CDC Estimates	Obesity (95% CI) CHOICES Model	Severe Obesity (95% CI) CHOICES Model
Alabama	32.4 (30.8–34.1)	38.8 (36.9–40.8)	18.1 (16.6–19.7)
Alaska	28.4 (26.5–30.4)	34.6 (32.4–36.8)	13.4 (11.8–15)
Arizona	26.8 (24.3–29.4)	31.3 (28.7–33.9)	11.0 (9.3–12.7)
Arkansas	34.6 (32.7–36.6)	40.5 (38.4–42.7)	17.4 (15.8–19.1)
California	24.1 (23.0–25.3)	28.8 (27.6–30.1)	10.9 (10.1–11.7)
Colorado	21.3 (20.4–22.2)	26.0 (25.0–27.1)	9.5 (8.8–10.2)
Connecticut	25.0 (23.5–26.4)	30.6 (28.9–32.2)	11.4 (10.3–12.5)
Delaware	31.1 (29.3–32.8)	36.1 (34.1–38.0)	15.3 (13.8–16.7)
District of Columbia	22.9 (21.0–24.8)	27.2 (24.9–29.4)	12.2 (10.6–13.8)
Florida	26.4 (25.3–27.4)	33.3 (32.0–34.5)	12.8 (11.9–13.7)
Georgia	30.3 (28.9–31.8)	35.5 (33.9–37.1)	15.7 (14.5–16.9)
Hawaii	21.8 (20.4–23.2)	22.9 (21.4–24.3)	7.7 (6.9–8.6)
Idaho	29.6 (27.8–31.4)	34.9 (32.9–36.9)	13.5 (12.1–15)
Illinois	29.4 (27.7–31.2)	34.9 (33.0–36.8)	14.3 (12.9–15.6)
Indiana	31.8 (30.6–33.1)	37.8 (36.4–39.2)	16.5 (15.5–17.6)
Iowa	31.3 (29.9–32.7)	37.7 (36.2–39.2)	15.8 (14.7–17.0)
Kansas	30.0 (29.2–30.7)	35.7 (34.8–36.5)	15.3 (14.7–16.0)
Kentucky	33.2 (31.8–34.6)	39.4 (37.7–41.1)	17.3 (16.0–18.6)
Louisiana	33.1 (31.1–35.2)	37.5 (35.2–39.7)	17.5 (15.7–19.2)
Maine	28.9 (27.5–30.2)	34.9 (33.4–36.4)	14.0 (12.9–15.1)
Maryland	28.3 (27.0–29.5)	33.0 (31.6–34.4)	14.3 (13.2–15.3)
Massachusetts	23.6 (22.5–24.8)	28.9 (27.6–30.2)	11.1 (10.2–12.0)
Michigan	31.5 (30.4–32.6)	38.1 (36.8–39.4)	16.5 (15.6–17.5)
Minnesota	25.5 (24.1–26.8)	31.7 (30.2–33.3)	12.0 (10.9–13.0)
Mississippi	35.1 (33.5–36.8)	40.5 (38.7–42.3)	19.0 (17.5–20.4)
Missouri	30.4 (28.8–32.1)	35.8 (33.8–37.9)	15.4 (13.8–16.9)
Montana	24.6 (23.4–25.8)	30.0 (28.7–31.4)	11.4 (10.5–12.3)
Nebraska	29.6 (28.4–30.7)	35.3 (34.1–36.6)	15.1 (14.1–16.0)
Nevada	26.2 (24.0–28.6)	31.5 (29.0–34.1)	12.3 (10.4–14.1)
New Hampshire	26.7 (25.3–28.3)	33.0 (31.3–34.7)	12.7 (11.6–13.9)
New Jersey	26.3 (25.1–27.5)	32.7 (31.3–34.1)	11.9 (10.9–12.9)
New Mexico	26.4 (25.1–27.7)	31.5 (30.0–33.0)	11.4 (10.5–12.4)
New York	25.4 (24.2–26.6)	30.9 (29.4–32.4)	12.0 (11.0–13.0)
North Carolina	29.4 (28.1–30.7)	35.7 (34.2–37.3)	15.8 (14.6–17.0)
North Dakota	31.0 (29.5–32.5)	37.1 (35.4–38.8)	15.3 (14.0–16.6)
Ohio	30.4 (29.2–31.6)	36.8 (35.4–38.2)	15.5 (14.5–16.6)
Oklahoma	32.5 (31.2–33.9)	38.7 (37.2–40.2)	16.6 (15.5–17.7)
Oregon	26.5 (24.9–28.1)	32.4 (30.6–34.1)	13.1 (11.8–14.4)
Pennsylvania	30.0 (28.9–31.2)	36.3 (35.0–37.6)	14.9 (13.9–15.9)
Rhode Island	27.3 (25.8–28.8)	33.4 (31.7–35.1)	13.4 (12.1–14.6)
South Carolina	31.7 (30.5–33.1)	37.9 (36.5–39.4)	16.5 (15.3–17.6)
South Dakota	29.9 (28.0–31.8)	35.3 (33.1–37.4)	14.6 (13.0–16.1)
Tennessee	33.7 (31.9–35.5)	40.5 (38.2–42.7)	17.3 (15.5–19.0)
Texas	30.9 (29.5–32.3)	35.4 (33.7–37.1)	15.5 (14.2–16.8)
Utah	24.1 (23.2–25.1)	30.6 (29.4–31.7)	11.8 (11.1–12.6)
Vermont	24.7 (23.4–26.1)	31.3 (29.7–32.9)	12.1 (11.0–13.2)
Virginia	27.2 (25.9–28.5)	32.7 (31.3–34.2)	12.9 (11.9–13.9)
Washington	27.2 (26.0–28.3)	31.8 (30.5–33.0)	13.6 (12.6–14.5)
West Virginia	35.1 (33.6–36.6)	40.8 (39.1–42.4)	17.6 (16.4–18.9)
Wisconsin	29.8 (28.0–31.6)	35.5 (33.5–37.5)	14.3 (12.9–15.8)
Wyoming	27.8 (26.2–29.5)	32.9 (31.1–34.7)	13.1 (11.8–14.4)

## Discussion

While the existing maps and prevalence estimates based on self-reported data have been useful in highlighting trends in obesity, bias in self-reported height and weight causes current CDC maps to substantially underestimate state-specific obesity prevalence in the US. Although these maps have been critical tools for the public health community in raising awareness about the state-level burden of obesity, their lack of accuracy limits the ability of state policymakers to base obesity prevention policies on accurate state-level estimates of obesity-related mortality, morbidity, or healthcare costs.

Previous regression-based efforts go some way to addressing self-report bias. However, as the results of this study show, although regression adjustment produces reasonably accurate estimates of mean BMI, it still significantly underestimates national obesity prevalence. Since regression works by estimating the *average* value of the dependent variable, the resulting distribution of BMI is thus concentrated around the expected value [15]. This shrinking of the distribution tails is especially problematic for producing prevalence estimates of severe obesity, a condition associated with substantially increased risks of morbidity, mortality, and health services utilization [31]. The economic implications of undercounting millions of cases of obesity are large. For example, assuming incremental obesity-related healthcare costs of $1,000 per individual (which is likely a conservative estimate [31–33]), undercounting 12 million cases of obesity would result in missing $12 billion of costs. Regression-adjusted estimates would still miss $2–3 billion of healthcare costs.

In contrast, we have shown that our statistical matching approach preserves the entire BMI distribution while correcting for self-report bias. This approach accounts for the geographic variation in self-reported obesity while yielding valid national-level estimates compared to NHANES data. To our knowledge, no other adjustment method has been validated against measured data. The corrected 2013 estimates of state-specific obesity (Fig 1b) and severe obesity (Fig 2b) paint a more accurate picture of the obesity epidemic, and highlight how small biases in individual-level BMI can result in substantial shifts in population-level prevalence estimates.

In addition, statistical matching is flexible with respect to variables of interest, and other datasets. Individual-level matching allows us to control for differential self-report bias by salient factors such as race/ethnicity and income, thus capturing any latent obesity gradients with respect to the matched variables. The approach is also extensible to multiple datasets, allowing the CHOICES model to synthesize information from a range of sources to create a richer virtual population. As a reproducible, computationally feasible method, it is also straightforward to update estimates as newer data become available.

### Limitations

Although statistical matching is a powerful approach, it is not without limitations. To increase sample size, we pooled NHANES data from 2007–2012, which did not allow us to model trends that may have occurred within this period. However, we found that mean BMI and obesity did not change significantly over this period (data not shown), suggesting that pooling these years did not substantially bias our estimates. Similarly, we found no significant change in self-report bias over this period, suggesting that the percentile calculations of self-reported data were largely unaffected by pooling. However, the potential for differential or secular trends to bias the results highlights the tension between increasing sample size and the validity of pooling data across time periods.

Although past rounds of BRFSS reported age in single years, the 2013 dataset only reports 5-year age groups, with the lowest group collapsed across 18–24 year olds and age top-coded at 80. We therefore used the midpoint of each age group to match individuals in BRFSS to those in NHANES. While these broader age groups limited the precision of the matching process, the resulting estimates of BMI distributions within sex-specific age groups were similar to observed distributions in NHANES (see Table C in S1 File).

While our approach controlled for geographic variation in self-report bias due to demographic composition, it did not eliminate potential residual variation within subgroups. A recent paper by Le et al. reported differential self-report bias in obesity prevalence by region based on a comparison of self-reported height and weight from BRFSS and NHANES within Census regions [34]. However, because the authors focused on obesity prevalence rather than BMI, it is unclear whether the observed variation was due to actual regional differences in self-report bias, or was simply the result of different underlying BMI distributions across regions. As we have shown, the effect of self-report bias on obesity prevalence varies greatly depending on the location of the underlying BMI distribution relative to the specific cut-point used; estimates for states with high obesity prevalence are generally less sensitive to adjustments for self-report bias since a bulk of the self-reported BMI distribution is already over 30. While we cannot rule out residual regional variation in self-report bias, the matching methods used were applied within demographic strata (defined by age, sex, race/ethnicity, and household income), so we eliminated any regional variation in self-report bias due to compositional differences in these factors. Future studies could improve upon these methods by matching BRFSS to restricted regional NHANES data, although the smaller sample size within regions may be an issue.

## Conclusions

The corrected estimates of adult obesity reveals that in many states, the obesity epidemic is worse than previously reported. Although self-report bias has been well-documented, the extent to which it affects population-level estimates of obesity has not always been fully appreciated. The argument that “everybody knows” that state-level estimates based on self-reported data are too low is of little help in actually producing defensible estimates which are necessary for any realistic analysis aimed to inform policy. Knowingly underestimating millions of cases of obesity and billions of dollars of associated costs is a misleading exercise.

While commonly used regression-based approaches can mitigate the effects of self-report bias, they still result in underestimates of obesity prevalence. In contrast, we have shown that non-parametric statistical matching can generate valid national estimates of obesity prevalence compared to measured data while retaining the state-level variations observed in self-reported data. Accurate state-specific obesity estimates are necessary to help officials plan appropriately for the medical capacity and economic resources needed to address this epidemic, and institute preventive measures where they are needed most.

## Supporting Information

S1 File Table A, Self-reported vs measured height and weight in NHANES 2007–2012 and BRFSS 2013. Table B, Dataset crosswalks for matching individuals from BRFSS to NHANES. Table C, Dynamic subgroup definitions. Table D, Individual-level linear regression of measured height and weight on self-reported data in NHANES 2007–2012. Table E, Aggregate-level comparison of measured mean BMI in NHANES 2007–2012 to self-reported mean BMI from BRFSS 2013. Table F, Two-sample Kolmogorov-Smirnov tests comparing age- and sex-specific BMI distributions from NHANES to BRFSS by adjustment method.
(PDF)
