# Supplementary material for: Redrawing the US Obesity Landscape: Bias-Corrected Estimates of State-Specific Adult Obesity Prevalence
Source: PLoS One. 2016 Mar 8;11(3):e0150735. doi: 10.1371/journal.pone.0150735 (PMC4782996; doi:10.1371/journal.pone.0150735)
Supplement: S1 File — Table A, Self-reported vs measured height and weight in NHANES 2007–2012 and BRFSS 2013. Table B, Dataset crosswalks for matching individuals from BRFSS to NHANES. Table C, Dynamic subgroup definitions. Table D, Individual-level linear regression of measured height and weight on self-reported data in NHANES 2007–2012. Table E, Aggregate-level comparison of measured mean BMI in NHANES 2007–2012 to self-reported mean BMI from BRFSS 2013. Table F, Two-sample Kolmogorov-Smirnov tests comparing age- and sex-specific BMI distributions from NHANES to BRFSS by adjustment method. (PDF) [file pone.0150735.s001.pdf]

Supplementary Appendix to "Redrawing the US Obesity Landscape: Bias-Corrected Estimates of State-Specific Adult Obesity Prevalence"

Zachary J. Ward[1]*, Michael W. Long[2], Stephen C. Resch[1], Steven L. Gortmaker[3], Angie L. Cradock[3], Catherine Giles[3], Amber Hsiao[4], Y. Claire Wang[4]

1. Center for Health Decision Science, Harvard T.H. Chan School of Public Health, Boston, MA, USA
2. Department of Prevention and Community Health, Milken Institute School of Public Health, the George Washington University, Washington DC, USA
3. Department of Social and Behavioral Sciences, Harvard T.H. Chan School of Public Health, Boston, MA, USA
4. Department of Health Policy and Management, Mailman School of Public Health, Columbia University, New York, NY, USA

* Corresponding author
Email: zward@hsph.harvard.edu (ZJW)

Contents:

Table A: Self-reported vs measured height and weight in NHANES 2007-2012 and BRFSS 2013

Table B: Dataset crosswalks for matching individuals from BRFSS to NHANES

Table C: Dynamic subgroup definitions

Table D: Individual-level linear regression of measured height and weight on self-reported data in NHANES 2007-2012

Table E: Aggregate-level comparison of measured mean BMI in NHANES 2007-2012 to self-reported mean BMI from BRFSS 2013

Table F: Two-sample Kolmogorov-Smirnov tests comparing age- and sex-specific BMI distributions from NHANES to BRFSS by adjustment method

Table A: Self-reported vs measured height and weight in NHANES 2007-2012 and BRFSS 2013

1. Paired t-tests: NHANES self-report vs measured – within individuals (18+)[a]

HEIGHT (cm)	Measured Mean (95% CI)	Self-Report Mean (95% CI)	df	t	*P* Value
Males	176.01 (175.68-176.34)	177.35 (177.00-177.69)	8471	38.35	*P*<.001
Females	162.19 (161.90-162.47)	163.12 (162.86-163.38)	8451	31.87	*P*<.001
**WEIGHT (kg)**					
Males	88.34 (87.61-89.06)	88.23 (87.51-88.96)	8471	-1.42	.16
Females	75.11 (74.49-75.73)	73.78 (73.19-74.37)	8451	-25.67	*P*<.001
**BMI**					
Males	28.45 (28.22-28.69)	28.00 (27.78-28.22)	8471	-19.69	*P*<.001
Females	28.54 (28.32-28.75)	27.71 (27.51-27.92)	8451	-37.62	*P*<.001

2. Two-sample t-tests (sample-weighted): NHANES self-report (in-person interview) vs BRFSS self-report (telephone interview) – adults 18+[b]

HEIGHT (cm)	NHANES Mean (95% CI)	BRFSS Mean (95% CI)	df	t	*P* Value
Males	177.35 (177.00-177.69)	177.82 (177.74-177.90)	176 702	3.97	*P*<.001
Females	163.12 (162.86-163.38)	163.28 (163.21-163.34)	227 013	1.44	.15
**WEIGHT (kg)**					
Males	88.23 (87.51-88.96)	88.62 (88.44-88.81)	176 702	1.38	.17
Females	73.78 (73.19-74.37)	73.50 (73.33-73.68)	227 013	-1.01	.31
**BMI**					
Males	28.00 (27.78-28.22)	27.98 (27.92-28.03)	176 702	-0.33	.74
Females	27.71 (27.51-27.92)	27.56 (27.50-27.62)	227 013	-1.55	.12

[a] Height is overestimated by self-report, while weight is underestimated by females, leading to a significant difference in self-reported vs measured BMI for both sexes.
[b] A comparison of in-person self-report vs telephone self-report revealed a small, significant difference for male height, but no significant differences for weight or BMI for either sex.

Table B: Dataset crosswalks for matching individuals from BRFSS to NHANES

Race/Ethnicity Category Crosswalks

<table>
<tr><th>BRFSS Race Category</th><th>NHANES Race Category</th></tr>
<tr><td>White, Non-Hispanic</td><td>White, Non-Hispanic</td></tr>
<tr><td>Black or African American, Non-Hispanic</td><td>Black, Non-Hispanic</td></tr>
<tr><td>American Indian and Alaska Native, Non-Hispanic</td><td rowspan="5">Other Race, including Multi-Racial</td></tr>
<tr><td>Asian, Non-Hispanic</td></tr>
<tr><td>Native Hawaiian and Other Pacific Islander, Non-Hispanic</td></tr>
<tr><td>Other, Non-Hispanic</td></tr>
<tr><td>Two or more races, Non-Hispanic</td></tr>
<tr><td rowspan="2">Hispanic</td><td>Mexican American</td></tr>
<tr><td>Other Hispanic</td></tr>
</table>

Income Category Crosswalks

<table>
<tr><th>BRFSS Income Category</th><th colspan="3">NHANES Income Categories</th></tr>
<tr><td rowspan="2"><$10,000</td><td>$0-$4,999</td><td rowspan="4"><$20,000</td><td rowspan="11"></td></tr>
<tr><td>$5,000-$9,999</td></tr>
<tr><td>$10,000-$14,999</td><td>$10,000-$14,999</td></tr>
<tr><td>$15,000-$19,999</td><td>$15,000-$19,999</td></tr>
<tr><td>$20,000-$24,999</td><td>$20,000-$24,999</td><td rowspan="9">>$20,000</td></tr>
<tr><td>$25,000-$34,999</td><td>$25,000-$34,999</td></tr>
<tr><td rowspan="2">$35,000-$49,999</td><td>$35,000-$44,999</td></tr>
<tr><td rowspan="2">$45,000-$54,999</td></tr>
<tr><td rowspan="3">$50,000-$74,999</td></tr>
<tr><td>$55,000-$64,999</td></tr>
<tr><td>$65,000-$74,999</td></tr>
<tr><td rowspan="2">$75,000 or more</td><td>$75,000-$99,999</td><td rowspan="2">>$75,000</td></tr>
<tr><td>>$100,000</td></tr>
</table>

Table C: Dynamic subgroup definitions

Iteration	Matching Group	Cumulative % Matched
0	(Exact Match)	35.08%
1	Income Range 1	71.51%
2	Income Range 2	82.32%
3	Income Range 3	85.34%
4	Income Range 4	86.95%
5	Income Range 5	87.72%
6	Income Range 6	87.85%
7	Age Range 1	98.07%
8	Age Range 2	99.53%
9	Age Range 3	99.89%
10	Age Range 4	100%

Table D: Individual-level linear regression[a] of measured height and weight on self-reported data in NHANES 2007-2012

Males – Weight

	White		Black		Hispanic		Other	
**Coefficient**	**Value**	***P* value**	**Value**	***P* value**	**Value**	***P* value**	**Value**	***P* value**
Self-reported weight (lbs)	0.9691	*P*<.001	1.0814	*P*<.001	0.9324	*P*<.001	0.8352	*P*<.001
Self-reported weight (lbs) squared	0.0001	.26	-0.00004	.87	0.0002	.21	0.0005	.07
Age in years	-0.0607	.25	-0.0834	.50	0.0799	.38	0.1996	.13
Age in years squared	0.0004	.40	0.0001	.94	-0.0013	.17	-0.0024	.08
Constant	2.8965	.54	-11.6101	.29	5.3957	.36	8.7056	.40
Observations	3812		1851		2053		756	
R-squared	0.9618		0.9347		0.9319		0.9609	

Females-Weight

	White		Black		Hispanic		Other	
**Coefficient**	**Value**	***P* value**	**Value**	***P* value**	**Value**	***P* value**	**Value**	***P* value**
Self-reported weight (lbs)	1.1228	*P*<.001	1.0567	*P*<.001	1.1301	*P*<.001	1.0520	*P*<.001
Self-reported weight (lbs) squared	-0.0003	.02	-0.00004	.75	-0.0003	*P*<.001	-0.0001	.31
Age in years	0.0273	.55	0.0233	.81	-0.0915	.23	0.1028	.32
Age in years squared	-0.0007	.13	-0.0012	.21	0.0006	.47	-0.0016	.14
Constant	-9.2384	.004	-2.9394	.56	-8.4144	*P*<.001	-4.9116	.16
Observations	3694		1901		2135		722	
R-squared	0.9646		0.9389		0.9474		0.9652	

Males – Height

	White		Black		Hispanic		Other	
**Coefficient**	**Value**	***P* value**	**Value**	***P* value**	**Value**	***P* value**	**Value**	***P* value**
Self-reported height (in)	0.1209	.79	0.3930	.40	-2.3019	*P*<.001	-1.4057	.21
Self-reported height (in) squared	0.0054	.09	0.0031	.35	0.0226	*P*<.001	0.0165	.045
Age in years	0.0415	*P*<.001	0.0445	*P*<.001	0.0374	.001	0.0529	.002
Age in years squared	-0.0006	*P*<.001	-0.0006	*P*<.001	-0.0006	*P*<.001	-0.0007	*P*<.001
Constant	33.7789	.03	26.3059	.11	118.9499	*P*<.001	86.2105	.02
Observations	3812		1851		2053		756	
R-squared	0.8834		0.8691		0.7639		0.8631	

Table D: Individual-level linear regression[a] of measured height and weight on self-reported data in NHANES 2007-2012 (continued)

Females – Height

	White		Black		Hispanic		Other	
**Coefficient**	**Value**	***P* value**	**Value**	***P* value**	**Value**	***P* value**	**Value**	***P* value**
Self-reported height (in)	1.6254	.001	3.1089	*P*<.001	-2.8043	*P*<.001	0.0200	.99
Self-reported height (in) squared	-0.0058	.12	-0.0178	.002	0.0284	*P*<.001	0.0068	.56
Age in years	0.0385	*P*<.001	0.0583	*P*<.001	0.0136	.15	0.0138	.30
Age in years squared	-0.0006	*P*<.001	-0.0008	*P*<.001	-0.0004	*P*<.001	-0.0003	.03
Constant	-16.9758	.26	-62.8755	.008	126.2547	*P*<.001	34.3916	.46
Observations	3694		1901		2135		722	
R-squared	0.904		0.8212		0.7337		0.8511	

SI conversion factors: To convert inches to centimeters, multiply values by 2.54. To convert pounds to kilograms, multiply values by 0.45.

[a] Linear regression models are based on Cawley J, Burkhauser R. Beyond BMI: The Value of More Accurate Measures of Fatness and Obesity in Social Science Research. NBER Working Paper Series. 2006;Working Paper 12291.

Table E: Aggregate-level comparison[a] of measured mean BMI in NHANES 2007-2012 to self-reported mean BMI from BRFSS 2013

Sex – Age Group	NHANES Mean BMI (Measured)	BRFSS Mean BMI (Self-Report)
**Males – 18-34**	27.10861	26.56635
**Males – 35-44**	29.24743	28.73203
**Males – 45-54**	29.20621	28.91157
**Males – 55-64**	29.17282	28.80128
**Males – 65-74**	29.09742	28.36223
**Males – 75+**	27.6745	26.85956
**Females – 18-34**	27.42154	26.29227
**Females – 35-44**	28.57631	28.0812
**Females – 45-54**	29.19066	28.175
**Females – 55-64**	29.65947	28.49906
**Females – 65-74**	29.49242	28.19293
**Females – 75+**	27.33292	26.58255

[a] Method is from Dwyer-Lindgren L, Freedman G, Engell RE, et al. Prevalence of physical activity and obesity in US counties, 2001-2011: a road map for action. *Population Health Metrics*. 2013;11:7. Note that in the original implementation, linear regression was used to adjust mean BMI over different years of NHANES and BRFSS. As we are simply looking at one year of BRFSS data, this regression approach resolves to simply applying a scalar to self-reported data (i.e. no intercept).

Table F: Two-sample Kolmogorov-Smirnov tests comparing age- and sex-specific BMI distributions from NHANES to BRFSS by adjustment method

MALES	BRFSS (Unadjusted)			Individual-level Regression[a]			Aggregate-level Regression[b]			CHOICES Model (Statistical Matching)		
Age Group	D[c]	*P* Value	BMI[d]	D[c]	*P* Value	BMI[d]	D[c]	*P* Value	BMI[d]	D[c]	*P* Value	BMI[d]
**18-24**	0.0807	*P*<.001	28.2	0.0579	.001	28.5	0.0471	.01	28.2	0.0331*	.10	30.0
**25-29**	0.0882	*P*<.001	28.1	0.0675	.005	29.1	0.0635	.009	24.7	0.0357*	.22	24.5
**30-34**	0.0540	.03	25.8	0.0312*	.30	26.3	0.0438*	.09	25.4	0.0344*	.23	28.2
**35-39**	0.0706	.001	30.8	0.0445*	.08	30.8	0.0460*	.06	23.3	0.0428*	.09	35.9
**40-44**	0.0773	.001	25.1	0.0521	.03	25.2	0.0320*	.28	28.7	0.0185*	.65	24.3
**45-49**	0.0639	.006	28.3	0.0456*	.08	28.3	0.0492	.049	25.1	0.0313*	.29	23.5
**50-54**	0.0639	.003	25.8	0.0333*	.21	32.8	0.0456*	.052	26.1	0.0277*	.34	25.0
**55-59**	0.0428*	.13	24.1	0.0542	.04	24.1	0.0626	.01	24.5	0.0324*	.31	27.0
**60-64**	0.0691	.001	30.1	0.0349*	.16	30.0	0.0409*	.08	30.1	0.0273*	.33	28.6
**65-69**	0.0826	.001	31.6	0.0650	.01	24.5	0.0798	*P*<.001	25.6	0.0383*	.20	35.9
**70-74**	0.1230	*P*<.001	27.4	0.0575	.049	27.4	0.0543*	.07	27.4	0.0364*	.30	24.9
**75-79**	0.1209	*P*<.001	29.2	0.0525*	.13	23.9	0.0523*	.13	23.9	0.0549*	.11	30.5
**80+**	0.1095	*P*<.001	25.8	0.0256*	.54	29.7	0.0397*	.23	28.3	0.0363*	.29	29.1

FEMALES	BRFSS (Unadjusted)			Individual-level Regression[a]			Aggregate-level Regression[b]			CHOICES Model (Statistical Matching)		
Age Group	D[c]	*P* Value	BMI[d]	D[c]	*P* Value	BMI[d]	D[c]	*P* Value	BMI[d]	D[c]	*P* Value	BMI[d]
**18-24**	0.1049	*P*<.001	28.3	0.0705	*P*<.001	30.2	0.0785	*P*<.001	20.5	0.0379*	.07	29.2
**25-29**	0.0756	.001	29.3	0.0461*	.09	35.7	0.0393*	.17	22.5	0.0337*	.27	28.8
**30-34**	0.0873	*P*<.001	23.8	0.0480*	.06	23.8	0.0323*	.27	27.6	0.0394*	.14	23.8
**35-39**	0.0824	*P*<.001	26.6	0.0442*	.08	31.6	0.0563	.02	30.7	0.0222*	.52	19.9
**40-44**	0.0463*	.052	25.9	0.0509	.03	23.7	0.0394*	.12	23.7	0.0344*	.20	33.5
**45-49**	0.0925	*P*<.001	28.3	0.0418*	.09	28.3	0.0345*	.20	37.0	0.0353*	.18	28.1
**50-54**	0.0926	*P*<.001	28.3	0.0416*	.09	23.9	0.0331*	.22	28.5	0.0446*	.06	25.0
**55-59**	0.0755	.002	32.3	0.0449*	.11	22.3	0.0627	.01	22.4	0.0235*	.54	39.7
**60-64**	0.1156	*P*<.001	27.5	0.0534	.01	28.7	0.0528	.02	28.6	0.0294*	.27	23.8
**65-69**	0.0790	.002	33.2	0.0412*	.18	22.1	0.0620	.02	22.0	0.0499*	.08	28.8
**70-74**	0.1533	*P*<.001	27.5	0.1002	*P*<.001	32.3	0.0881	*P*<.001	33.2	0.0389*	.22	34.0
**75-79**	0.1352	*P*<.001	29.3	0.0612*	.07	29.8	0.0856	.006	29.3	0.0304*	.52	25.0
**80+**	0.0734	.003	26.6	0.0680	.008	32.3	0.0423*	.15	32.4	0.0272*	.46	26.8

[a] Individual-level regression models are based on Cawley J, Burkhauser R. Beyond BMI: The Value of More Accurate Measures of Fatness and Obesity in Social Science Research. NBER Working Paper Series. 2006;Working Paper 12291.
[b] Aggregate-level regression adjustment is based on Dwyer-Lindgren L, Freedman G, Engell RE, et al. Prevalence of physical activity and obesity in US counties, 2001-2011: a road map for action. *Population Health Metrics*. 2013;11:7.
[c] The D-Statistic is the maximum vertical distance between the cumulative distributions.
[d] BMI indicates at what point in the cumulative BMI distributions the D-Statistic occurs.
* BMI distributions are not statistically different from NHANES (p>.05).
